# A mechanistically informed framework of hierarchical temporal speech organization for objective differential assessment of motor speech disorders in neurodegenerative diseases

**DOI:** 10.3389/fnins.2026.1907826

**Published:** 2026-09-04

**Authors:** Kaley Bruning, Lindsey Heidrick, Panying Rong

**Affiliations:** 1 Department of Speech-Langue-Hearing: Sciences and Disorders, University of Kansas, Lawrence, KS, United States; 2 Department of Hearing and Speech, University of Kansas Medical Center, Kansas City, KS, United States

**Keywords:** acoustics, articulatory kinematics, differential assessment, motor speech disorder, neurodegenerative disease, objective marker, pause, rhythm

## Abstract

**Introduction:**

Speech is organized into discrete utterances and pauses in accordance with underlying linguistic structure, temporal coordination, prosodic organization, and respiratory demands. Temporal speech characteristics, including rhythmic and pausing patterns, are governed by multiple neurophysiological mechanisms and provide valuable insights for the assessment and management of motor speech disorders. This study employed a mechanistically informed analytic framework of hierarchical temporal speech organization to derive interpretable, objective measures for assessing rhythmic and pausing disturbances in neurodegenerative diseases.

**Methods:**

Orofacial kinematic and acoustic recordings were obtained from two neurodegenerative disease groups—amyotrophic lateral sclerosis (ALS) and Parkinson's disease (PD)—along with a neurologically healthy control (HC) group, during a passage reading task. Using automated analytic procedures, kinematic rhythm measures (movement-based) and acoustic rhythm measures (sound-based) were derived to characterize hierarchical rhythmic modulation of articulatory movements and critical-band acoustic modulation envelopes at prosodic, syllabic, and sub-syllabic levels. Pause measures were derived to characterize within- and between-sentence pausing patterns. Rhythmic and pausing patterns were compared across groups. The acoustically derived rhythm and pause measures were subsequently subjected to (1) discriminant analyses for multiclass classification among the ALS, PD, and HC groups, and (2) regularized regression models to evaluate their associations with functional outcomes.

**Results:**

Both neurodegenerative disease cohorts exhibited a reorganization of hierarchical rhythmic modulation characterized by reduced prosodic-level modulation and increased syllabic-level modulation. Such changes were, however, driven primarily by articulatory impairment in ALS and by respiratory-laryngeal impairment in PD. In addition, both cohorts showed reduced regularity of intra-syllabic temporal organization and altered within-sentence pausing patterns, whereas the ALS cohort exhibited additional changes in between-sentence pausing. Measures capturing these rhythmic and pausing disturbances demonstrated promising classification performance (mean accuracy = 0.77; mean area under the curve [AUC] = 0.84) and meaningful associations with functional speech decline across diseases.

**Discussion:**

The rhythm and pause measures captured disease-specific subclinical changes in the physiological substrates underlying rhythmic and pausing disturbances in ALS and PD, demonstrating potential as clinically applicable, objective markers to enable more accurate differential diagnosis, targeted intervention, and measurement-based care for neurodegenerative motor speech disorders.

## 1 Introduction

Neurodegenerative diseases are progressive disorders that present distinct yet overlapping clinicopathological features, with profound heterogeneity within disease (Brettschneider et al., 2015). The pathophysiology of neurodegenerative diseases is associated with the dynamic spreading of pathological proteins across neural networks, leading to ongoing degeneration of neurons and other cells and, consequently, progressive declines in motor, cognitive, and behavioral functions (Spires-Jones et al., 2017). Motor speech disorders—particularly dysarthria—are highly prevalent in neurodegenerative diseases such as Parkinson's disease (PD) and amyotrophic lateral sclerosis (ALS). In PD, degeneration of dopaminergic neurons in the basal ganglia leads to motor impairments characterized by bradykinesia, rigidity, postural instability, and tremor. Motor speech impairment is among the most common manifestations of PD, affecting 70%−90% of individuals over the disease course (Moya-Gale and Levy, 2019; Ramig et al., 2008). The most prevalent form of motor speech disorder in PD is hypokinetic dysarthria, characterized by reduced and slowed movement that leads to monopitch, monoloudness, imprecise consonants, and harsh voice quality, as well as timing and rhythmic deficits, including reduced stress, inappropriate silences, short rushes of speech, and variable speech rate (Darley et al., 1969a,b). In ALS, degeneration of upper and lower motor neurons in the cerebrum, brainstem, and spinal cord leads to progressive weakness and a loss of voluntary control over the vast majority of skeletal muscles (Armon, 2008). Motor speech impairment—most commonly manifesting as mixed flaccid-spastic dysarthria—affects nearly all individuals diagnosed with bulbar-onset ALS (i.e., exhibiting speech and/or swallowing disorders as initial symptoms) and over 70% of individuals with spinal-onset ALS (i.e., exhibiting limb or other non-bulbar motor deficits as initial symptoms) over the disease course (da Costa Franceschini and Mourão, 2015; Donohue et al., 2023). The motor speech disorder associated with ALS is characterized by progressive neuromuscular weakness across speech physiological subsystems (i.e., articulatory, resonatory, laryngeal, respiratory) (Rong et al., 2015, 2016), leading to articulatory imprecision, hypernasality, and harsh voice quality, as well as impairments in coordinated subsystem actions, including monopitch, monoloudness, slow speech rate, short phrases, prolonged intervals, excess and equal stress, and inappropriate silences (Darley et al., 1969a,b).

Given the central role of communication in self-identity, social participation, and quality of life, early detection and timely, tailored management of motor speech disorders constitutes a critical component of clinical care in neurodegenerative diseases (Fried-Oken et al., 2015). Current clinical standards for differential assessment of motor speech disorders rely on a clinician-based evaluation framework that identifies abnormal auditory-perceptual features (e.g., imprecise consonants, monopitch) and clusters them into diagnostic profiles (Darley et al., 1969a,b; Duffy, 2013). However, this approach is largely subjective, time-intensive, and lacks sensitivity to subtle changes during the early disease stages. To address these limitations, there is a critical need for reliable, objective markers that (1) capture disease-specific motor speech impairments—including early-stage changes that may not yet be clinically detectable—to facilitate early detection and referral, and (2) are associated with meaningful clinical and functional changes to support targeted intervention planning.

Recent advances in signal analytics and artificial intelligence have accelerated the development of objective speech biomarkers and publicly available speech datasets, enabling increasingly sophisticated data-driven analyses of disordered speech (Berisha and Liss, 2024; Bhat and Strik, 2025; Bowden et al., 2023; Brahmi et al., 2024; Dubbioso et al., 2024a,b; Krautz et al., 2025; Martínez-Nicolás et al., 2021; Marx et al., 2026; Qian et al., 2023; Rong and Heidrick, 2026; Rusz et al., 2024; Sannino et al., 2026). In neurodegenerative diseases, a growing body of research has identified temporal speech characteristics, including rhythm and pausing patterns, as particularly informative indicators of motor speech impairments (Darling-White and Huber, 2020; Green et al., 2018, 2004; Huber and Darling, 2011; Huber et al., 2012; Liss et al., 2010, 2013, 2009; Rong, 2020; Rong and Heidrick, 2022, 2024; Rong and Liston, 2025; Rong and Rasmussen, 2024; Rong and Taylor, 2023; Yunusova et al., 2016, 2019). Pausing patterns reflect how speech is segmented into discrete temporal chunks, also referred to as utterances. These patterns are shaped by underlying linguistic structure, speech planning processes, prosodic emphasis, and respiratory coordination. Within utterances, speech is hierarchically organized across multiple timescales spanning the delta (0.9–2.5 Hz), theta (2.5–12 Hz), and beta/gamma (12–40 Hz) ranges, corresponding to the frequencies at which prosodic stress, syllables, and sub-syllabic units (e.g., onset rime/phoneme) recur, respectively, during natural speech (Leong and Goswami, 2015; Leong et al., 2017). Such phonological representations reflect the rhythmic entrainment of the speech production system to linguistic units at multiple levels, giving rise to hierarchical amplitude modulation patterns that enable the auditory system to parse incoming speech into prosodic, syllabic, and sub-syllabic units during speech comprehension (Giraud and Poeppel, 2012; Leong and Goswami, 2015; Riecke et al., 2018).

Impaired internal timing mechanisms—including interval timing, sequencing, predictive timing, and internal cueing—supported by central structures such as basal ganglia and cerebellum, as well as peripheral weakness arising from neuromuscular impairment, can all contribute to temporal disturbances in speech. Such disturbances manifest differently across disorders, resulting in (1) increased variability in speech timing and pause distribution, reduced stress, and short rushes of speech in PD (Duffy, 2013; Hammen and Yorkston, 1996; Skodda, 2011), (2) increased pauses, prolonged phonemes and intervals, and excess and equal stress in ALS (Duffy, 2013; Green et al., 2004; Yunusova et al., 2016), and (3) a percept of “scanning” speech, characterized by excess and equal stress, prolonged phoneme durations, and disrupted rhythmic timing in cerebellar ataxia (Ackermann and Hertrich, 1994; Duffy, 2013; Ziegler and Wessel, 1996), amongst other disorders. Collectively, these findings suggest that temporal speech disturbances may provide transdiagnostic insight into the integrity of neural motor control systems involved in speech production. Therefore, developing a reliable, objective method to detect, distinguish, and characterize these disturbances across diseases may provide valuable insights to facilitate early detection and differential assessment of motor speech disorders in neurodegenerative diseases.

In a series of prior studies, Rong and Heidrick developed a multimodal analytic framework to objectively assess and characterize hierarchical rhythmic organization in both kinematically and acoustically derived motor speech signals across delta, theta, and beta/gamma timescales (Rong and Heidrick, 2022, 2024). Those studies further demonstrated the utility of the framework for identifying rhythmic disturbances in ALS, which were primarily observed within the theta and beta/gamma timescales across both kinematic and acoustic modalities. These disturbances reflect disrupted rhythmic control of syllabic- and fine-scale articulatory and other physiological activities resulting from neuromuscular constraints. Additionally, speakers with ALS exhibited altered articulatory–acoustic relationships, characterized by a reallocation of articulatory resources to enhance articulatory contributions to syllabic timing, reflected in syllable-centered acoustic envelope modulation patterns. These patterns differ from those of healthy speakers, which exhibit distributed and specialized articulatory contributions to hierarchical rhythmic organization across prosodic, syllabic, and sub-syllabic levels (Rong and Heidrick, 2024).

The multimodal framework described above offers three major advantages that support its potential for clinical application. First, it is mechanistically informed by the multiscale temporal organization of speech, generating a set of interpretable, objective measures that characterize rhythmic control at prosodic, syllabic, and sub-syllabic levels, as well as the hierarchical organization of rhythmic timing across levels. Second, its multimodal design enables detailed characterization of the multifaceted nature of rhythmic control, which is governed by both articulatory activities (e.g., gestural excursion, sequencing, and coordination) and respiratory-laryngeal activities (e.g., intonation and loudness modulation, voice onset/offset timing). This characterization is achieved by dissociating the kinematic backbone of speech rhythm—reflected in rhythmic modulation patterns of individual articulatory movements—from the integrated acoustic outcome of rhythmic organization, represented by envelope modulation within critical spectral bands that encode distinct physiological processes (i.e., 100–300 Hz for vocal pitch and intonation; 300–800 Hz and 1,000–3,000 Hz for articulatory activities related to vowel formants; 3,000–8,000 Hz for articulatory activities related to high-frequency consonant noise). This approach enables comprehensive examination of both within- and between-subsystem coordination underlying rhythmic control. Given that articulatory and respiratory-laryngeal subsystems can be differentially affected across neurodegenerative diseases (Darley et al., 1969a,b), the multimodal framework has the potential to identify disease-specific physiological deficits contributing to rhythmic disturbances, thereby supporting differential assessment and more targeted intervention of motor speech disorders. Third, the analytic procedures are fully automated, enhancing the feasibility of clinical integration, particularly for objective measures derived from the acoustic modality.

Building upon this framework, the current study extends prior work by (1) integrating the rhythmic analysis with an additional pause analysis, and (2) applying this extended rhythm-pause analytic framework to two neurodegenerative disease cohorts—ALS and PD—to identify disease-specific patterns of rhythmic and pausing disturbances and to evaluate the utility of these measures for differential assessment of motor speech disorders. In addition, we examine associations between the objective rhythm and pause measures and standardized functional outcomes to evaluate their potential as objective markers of functional speech decline across diseases.

We hypothesize that individuals with ALS and PD will exhibit both shared and distinct patterns of rhythmic and pausing disturbances relative to healthy speakers, and that the combined rhythm-pause measures will detect and distinguish disease-specific motor speech impairments. Given existing clinical and research evidence showing articulatory-dominant impairments in ALS and respiratory-laryngeal-dominant impairments in PD, especially during early-to-moderate disease stages (Ramig et al., 2008; Rong et al., 2015; Skodda, 2011; Tjaden, 2008; Yorkston et al., 1993), it is expected that (1) rhythmic disturbances in ALS will be primarily driven by articulatory deficits, reflected in both kinematic rhythm measures and selective acoustic rhythm measures, particularly within the 300–800 Hz and/or 1,000–3,000 Hz spectral bands that encode vowel-related articulatory activities; and (2) rhythmic disturbances in PD will be pre-dominantly attributed to respiratory-laryngeal deficits, reflected in selective acoustic rhythm measures, particularly within the 100–300 Hz spectral band that encodes vocal pitch and intonation. In contrast, pausing behavior is expected to be altered in both ALS and PD. Furthermore, we expect to identify cross-disease objective markers of functional speech decline from the broader set of rhythm and pause measures.

## 2 Materials and methods

The study protocol was approved by the Institutional Review Board of the university medical center. Written informed consent was obtained from all participants.

### 2.1 Participants

Two neurodegenerative disease groups, including 16 individuals with ALS and 15 with PD, along with 10 neurologically healthy controls (HCs), participated in this study. Inclusion criteria for participants with neurodegenerative diseases included: (1) a diagnosis of ALS or PD by a certified neurologist; (2) native proficiency of American English; (3) passing hearing screening at 1,000, 2,000, and 4,000 Hz at 30 dB in at least one ear; (4) preserving oral communication capacity in daily life (i.e., not exclusively reliance on a speech generating device); (5) adequate cognitive capacity to understand and follow instructions; and (6) no reported depression or other psychiatric disorders. Healthy control participants reported no history of neurological disease or injury and met all other criteria above. Participants with neurodegenerative diseases were recruited from neurology clinics affiliated with the university health system, as well as local support and therapy groups. Healthy control participants were recruited from local communities.

All participants completed two standardized functional assessments: the Sentence Intelligibility Test (SIT) (Yorkston et al., 2007) and the Montreal Cognitive Assessment (MoCA) (Nasreddine et al., 2005). The SIT is administered using dedicated software that randomly generates 11 sentences with increasing length from 5 to 15 words, for a total of 110 words per participant. Participants read these sentences aloud from the computer screen using their natural speaking style. Speech was digitally recorded at a sampling rate of 22.05 kHz and subsequently orthographically transcribed and timed by two naïve listeners using the SIT software. The listeners are both native speakers of American English, with normal speech, language, hearing, and cognitive functions, and were unfamiliar with the stimuli and the speaker profiles. Based on the average responses between listeners, a standardized functional speech index—speech intelligibility—was calculated as the percentage of correctly transcribed words. MoCA is a recommended bedside cognitive screening instrument routinely used in clinical settings for neurodegenerative diseases (Strong et al., 2009). Cognitive impairment frequently co-occurs with motor speech impairment in neurodegenerative diseases and may confound certain speech measures, such as pausing patterns (Yunusova et al., 2016). Through a comprehensive assessment of cognitive domains—including executive function, memory, language, visuospatial function, attention, and concentration—the MoCA score provides an index of global cognitive function, enabling examination of potential confounding effects of cognitive impairment on the proposed rhythm and pause measures.

For participants with neurodegenerative diseases, a certified speech-language pathologist (the second author) conducted a structured auditory-perceptual evaluation of their SIT samples, using a checklist adapted from the Mayo system (Darley et al., 1969a,b; Duffy, 2013). This checklist, provided in Supplementary Table S1, consists of a comprehensive list of auditory-perceptual features that respectively assess articulatory, phonatory, resonatory, and respiratory subsystem performance, as well as constructs that require inter-subsystem coordination, including rate, rhythm, stress, and prosody. Each feature was assigned a binary score (0 = normal; 1 = abnormal), and the total score was calculated to index the overall severity of speech impairment. A summary of participant demographics and clinical characteristics is provided in Table 1.

**Table 1:** Participants characteristics.

Participant characteristics	ALS (*n* = 16)	PD (*n* = 15)	HC (*n* = 10)	Group comparisons
Demographic characteristics				
Women (*n* %)	37.50%	40.00%	70.00%	*p* = 0.25
Age, years (M; SD)	58.75; 14.58	69.87; 6.45	66.80; 13.02	
Clinical characteristics				
Disease duration, years since diagnosis (M; SD)	1.02; 1.11	4.36; 3.44	n.a.	n.a.
Disease onset (bulbar; spinal)	5; 11	n.a.	n.a.	n.a.
Speech impairment severity (M; SD)	3.44; 4.50	3.73; 3.01	n.a.	n.a.
MoCA score (M; SD)	25.56; 3.31	24.73; 4.20	26.75; 1.28	
Speech intelligibility, % (M; SD)	83.66; 30.15	94.90; 2.78	99.45; 0.56	

ALS, amyotrophic lateral sclerosis; PD, Parkinson's disease; HC, healthy control; Speech impairment severity, severity score derived from a clinician-based auditory-perceptual speech evaluation with a maximum score of 40; MoCA, Montreal Cognitive Assessment; WPM, words per minute; n.a., not applicable.

Group comparisons were conducted using Fisher's Exact Test for sex and Kruskal–Wallis Test for all other variables.

### 2.2 Experimental procedures

In the main experiment, participants read aloud the Rainbow Passage at their habitual speaking rate and loudness. This phonetically balanced passage consists of 19 sentences and 330 words, providing (1) comprehensive coverage of phonemes within the phonetic inventory of English to ensure the robustness of the analyses across linguistic content and (2) a sufficiently long connected speech sample for assessing rhythmic and pausing patterns representative of natural speech. During the reading task, kinematic and acoustic data were collected simultaneously using a 3D electromagnetic motion tracking system (Wave, Northern Digital Inc.) and a head-mounted microphone (DPA dfine 4188), following the protocol described in Rong and Heidrick (2024). All participants with PD were assessed during the on-medication state.

Prior to kinematic data collection, a midsagittal trace of the palate was acquired from the intersection between the hard and soft palates to central upper incisors, using a 3D probe supplied by Northern Digital Inc. This palatal trace provided a common oral anatomical reference for analyzing articulatory-kinematic data. Next, small, wired sensors were attached to five articulators, including (1) tongue tip (0.5–1 cm posterior to tongue apex), (2) tongue body (2–3 cm posterior to tongue tip), (3) lower lip (central vermillion boarder of lower lip), (4) upper lip (central vermillion boarder of upper lip), and (5) jaw (center of lower chin), using dental adhesive or medical tape. To minimize potential interference of the sensors with speech production, participants engaged in casual conversation with the experimenter and were encouraged to speak in their natural style for several minutes before data collection. This familiarization period allowed them to adapt to the sensors prior to the experimental task.

During data collection, articulatory movements were tracked by these sensors relative to a reference sensor embedded in a headband positioned at the center of the forehead, with the sensor axes aligned as closely as possible to the anatomical axes of the oral cavity. The resulting articulatory-kinematic data were recorded in 3D at a sampling rate of 100 Hz using the WaveFront software (Northern Digital Inc.). Acoustic data were acquired using the head-mounted microphone positioned approximately 5 cm from the left lip corner, conditioned through the Behringer Xenyx 802 sound mixer, and recorded simultaneously with the articulatory-kinematic data at a sampling rate of 22.05 kHz using the WaveFront software.

### 2.3 Data processing

The passage was segmented into utterances and pauses based on acoustic recordings. Pauses were operationally defined as silent intervals exceeding 150 ms in duration, whereas utterances were defined as continuous speech segments without pauses, regardless of the underlying linguistic structure. The temporal boundaries of all pause and utterance segments were identified and annotated by a trained analyst (the first author) using the TextGrid function in Praat (Boersma and Weenink, 2014). Pause segments were subsequently used to evaluate pausing patterns both within and between sentences. Temporal segments corresponding to utterances were extracted from both kinematic and acoustic recordings and concatenated for rhythmic analysis.

### 2.4 Rhythmic analysis

A detailed description of the analytic procedures for rhythmic analysis is provided in Rong and Heidrick (2024) and is briefly summarized below. All analyses were conducted at the sentence level.

#### 2.4.1 Kinematic rhythmic analysis

Focusing on the superior-inferior and anterior-posterior dimensions—which capture speech-related articulatory movements—kinematic recordings for the primary articulators, including tongue tip, tongue body, lower lip, and jaw, were low-passed filtered at 40 Hz using a second-order, zero-lag Butterworth filter. The filtered signals were then transformed to dissociate the passive loading effects of the jaw on the other articulators residing on it, using Equation 1 (Westbury et al., 2002):


$$\left[\begin{array}{l} x^{\prime }\\ y^{\prime } \end{array}]=[\begin{array}{cc} \cos\;\alpha & sin\alpha \\ -sin\alpha & cos\alpha \end{array}[[\begin{array}{c} \left(x-x_{j}\right)\\ \left(y-y_{j}\right) \end{array}\right]$$ (1)

where (*x*_*j*_, *y*_*j*_) are coordinates of the jaw sensor, (*x, y*) are the coordinates of the other articulatory sensors, (*x*′, *y*′) are the transformed coordinates of (*x, y*), and α is the jaw rotation angle estimated by the first principal component of jaw motion (*x*_*p*_) as α = 0.52*x*_*p*_.

Based on the transformed signals, the movement patterns of the tongue tip, tongue body, and lower lip were characterized by the Euclidian distances between the corresponding sensors and the jaw, yielding three time series representing tongue and lower lip movements independent of the jaw. The jaw movement pattern was characterized by the Euclidian distance between the jaw sensor and the anterior edge of the palatal trace, which corresponds to the location of central upper incisors. The resulting four articulatory time series were rate-normalized for each participant through linear time scaling based on the ratio of the participant's articulation rate to the mean articulation rate of the HC group. Here articulation rate was calculated as the total number of words produced (i.e., 330 words, adjusted for repetitions, self-corrections, and omissions) per unit of articulation time (i.e., total duration excluding pauses). This rate normalization was intended to control for potential confounding effects of rate variation on rhythmic patterns.

For each articulator, five rhythm measures were derived, including three modulation depths and two phase synchronization indices. To evaluate rhythmic modulation at prosodic, syllabic, and sub-syllabic levels, the power spectrum of each articulatory time series was calculated using a 2048-point Fast Fourier Transform (FFT) with a hamming window. Modulation depths within the delta, theta, and beta/gamma rhythmic ranges were derived as the proportion of spectral power within each target frequency range relative to total spectral power. To further evaluate temporal coupling between adjacent rhythms, each articulatory time series was decomposed into three oscillatory components using a fourth-order, zero-lag Butterworth filter with passbands of 0.9–2.5 Hz, 2.5–12 Hz, and 12–40 Hz. Based on these oscillatory signals, the phase synchronization index (PSI) was calculated to quantify how regularly fast oscillatory cycles are nested within the phase of the slow oscillatory signal, using Equation 2:


$$\begin{array}{c} PSI=|\langle e^{i\left(n\phi_{1}\left(t\right)-m\phi_{2}\left(t\right)\right)}\rangle | \end{array}$$ (2)

where ϕ_1_(*t*) and ϕ_2_(*t*) are the instantaneous phase of the oscillatory signals at time *t*; *n* and *m* are integers reflecting the frequency relation between the two oscillatory signals; and *nϕ*_1_(*t*)−*mϕ*_2_(*t*) represents the generalized phase difference, which is mapped to the [0,1] range, with higher values denoting greater synchrony. For each articulator, PSI was computed for the couplings between delta and theta rhythms as well as between theta and beta/gamma rhythms, with the ratio of *n*:*m* set to 2:1 and 3:1, respectively (Leong et al., 2017). Delta-theta PSI represents how regularly syllables are nested within the phase of prosodic units and can therefore be interpreted as an index of regularity of syllable stress. Theta-beta/gamma PSI reflects how regularly sub-syllabic units (e.g., vowels, consonants, and their transitions) are nested within the phase of syllables, which can be interpreted as indexing the regularity of intra-syllabic temporal organization.

### 2.5 Acoustic rhythmic analysis

The acoustic waveform was decomposed into 28 narrow-band signals distributed evenly along the cochlear frequency map between 100 and 10,000 Hz (Drullman, 1995; Smith et al., 2002). Such signals have been shown to generate envelop modulation patterns that closely correspond to those of speech-related physiological movements, such as lip aperture (Chandrasekaran et al., 2009). To derive the envelops, each narrow-band signal was subjected to Hilbert transformation. The resulting Hilbert envelopes were downsampled to 100 Hz, low-pass filtered at 40 Hz using a second-order, zero-lag Butterworth filter, and rate-normalized using the same linear time-scaling method applied in the kinematic rhythmic analysis. These narrow-band envelopes were then aggregated into four critical-band envelopes spanning the following spectral frequency ranges: (1) 100–300 Hz, which contains spectral content related to voicing and intonation; (2) 300–800 Hz, which corresponds primarily to the range of first formant frequencies of vowels; (3) 1,000–3,000 Hz, which reflects the range of second formant frequencies of vowels; and (3) 3,000–8,000 Hz, which captures high-frequency noise components related to consonants. These frequency ranges were selected because they correspond to the spectral bands in which the modulation patterns of acoustic envelopes exhibit the strongest correspondence with speech-related physiological movements across linguistically relevant timescales (Chandrasekaran et al., 2009).

In parallel with the kinematic rhythmic analysis, modulation depths within the delta, theta, and beta/gamma rhythmic ranges were derived from the power spectrum of each critical-band envelope as the proportion of spectral power within each target frequency range relative to total spectral power. To calculate PSI, each narrow-band envelope was decomposed into three oscillatory components using a fourth-order, zero-lag Butterworth filter with passbands of 0.9–2.5 Hz, 2.5–12 Hz, and 12–40 Hz. Oscillatory components corresponding to the same rhythmic range were aggregated across narrow bands within each critical band, yielding 12 critical-band signals (i.e., 3 oscillatory rhythms × 4 critical bands). Delta-theta PSI was calculated between critical-band signals oscillating within the delta and theta ranges, whereas theta-beta/gamma PSI was calculated between critical-band signals oscillating within the theta and beta/gamma ranges. A summary of all kinematic and acoustic rhythm measures is presented in Table 2.

**Table 2:** Summary of acoustic and kinematic rhythm measures.

Acoustic rhythm measure	Kinematic rhythm measure	Underlying construct
hbenvlp_mod_depth_delta_100_300	TT_mod_depth_delta	Delta modulation depth (prosodic-level rhythmic modulation)
hbenvlp_mod_depth_delta_300_800	TB_mod_depth_delta	
hbenvlp_mod_depth_delta_1000_3000	L_mod_depth_delta	
hbenvlp_mod_depth_delta_3000_8000	J_mod_depth_delta	
hbenvlp_mod_depth_theta_100_300	TT_mod_depth_theta	Theta modulation depth (syllabic-level rhythmic modulation)
hbenvlp_mod_depth_theta_300_800	TB_mod_depth_theta	
hbenvlp_mod_depth_theta_1000_3000	L_mod_depth_theta	
hbenvlp_mod_depth_theta_3000_8000	J_mod_depth_theta	
hbenvlp_mod_depth_beta.gamma_100–300	TT_mod_depth_beta.gamma	Beta/gamma modulation depth (sub-syllabic-level rhythmic modulation)
hbenvlp_mod_depth_beta.gamma_300_800	TB_mod_depth_beta.gamma	
hbenvlp_mod_depth_beta.gamma_1000_3000	L_mod_depth_beta.gamma	
hbenvlp_mod_depth_beta.gamma_3000_8000	J_mod_depth_beta.gamma	
hbenvlp_PSI_delta_theta_100_300	TT_PSI_delta_theta	Delta-theta phase synchronization index (regularity of syllable stress)
hbenvlp_PSI_delta_theta_300_800	TB_PSI_delta_theta	
hbenvlp_PSI_delta_theta_1000_3000	L_PSI_delta_theta	
hbenvlp_PSI_delta_theta_3000_8000	J_PSI_delta_theta	
hbenvlp_PSI_theta_beta.gamma_100_300	TT_PSI_theta_beta.gamma	Theta-beta/gamma phase synchronization index (regularity of intra-syllabic temporal organization)
hbenvlp_PSI_theta_beta.gamma_300_800	TB_PSI_theta_beta.gamma	
hbenvlp_PSI_theta_beta.gamma_1000_3000	L_PSI_theta_beta.gamma	
hbenvlp_PSI_theta_beta.gamma_3000_8000	J_PSI_theta_beta.gamma	

Hbenvlp, Hilbert envelope; TT, tongue tip; TB, tongue body; L, lower lip; J, jaw; PSI, phase synchronization index.

### 2.6 Pause analysis

Pause analysis was conducted at the passage level. Depending on the underlying linguistic structure, pauses identified throughout the entire passage were categorized as either within-sentence pauses or between-sentence pauses. For each category, total duration, mean duration, variability—quantified by the standard deviation (unnormalized variability) and coefficient of variation (normalized variability)—and percentage of pause duration were calculated to characterize within-and between-sentence pausing patterns. In addition, the total number of pauses across both categories was calculated as a global measure of pausing frequency. A summary of all pause measures is provided in Table 3.

**Table 3:** Summary of pause measures.

Pause measure	Underlying construct
TotDur_intrapause	Total pause duration within sentences
MeanDur_intrapause	Mean pause duration within sentences
SdevDur_intrapause	Standard deviation of pause duration within sentences
CvDur_intrapause	Coefficient of variation of pause duration within sentences
pct_intrapause	Percentage of pause time within sentences
TotDur_interpause	Total pause duration between sentences
MeanDur_interpause	Mean pause duration between sentences
SdevDur_interpause	Standard deviation of pause duration between sentences
CvDur_interpause	Coefficient of variation of pause duration between sentences
pct_interpause	Percentage of pause time between sentences
N_pause	Total number of pauses throughout the passage

### 2.7 Statistical analysis

Statistical analysis was conducted in the *R* Statistical Computing program (R Core Team, 2024). Prior to analysis, all rhythm and pause measures underwent standard data cleaning and imputation procedures. Data points falling outside the range of [lower quantile – 1.5^*^IQR, upper quantile + 1.5^*^IQR] were identified and inspected to determine whether they should be excluded as outliers. Missing data for repeated measures from different sentences were imputed using the two-level pan model (*2l.pan*), which accounts for within-subject dependence via multilevel modeling (Schafer and Yucel, 2002). Overall, 3.83% of observations for kinematically derived measures and 3.40% of observations for acoustically derived measures were imputed. Following data imputation, all rhythm measures were aggregated across the 19 sentences and then integrated with the pause measures to form a unified dataset for subsequent analyses.

#### 2.7.1 Disease-specific patterns of rhythmic and pausing disturbances

One-way analysis of variance (ANOVA) was used to evaluate the effect of group (3-level factor: ALS, PD, HC) on all rhythm and pause measures. To characterize disease-specific changes in rhythm and pausing patterns, *post-hoc* comparisons between each disease cohort and the HC group were conducted using estimated marginal means (Lenth, 2020), with effect sizes for between-group differences indexed by Cohen's d and *p-*values adjusted using the false discovery rate (FDR) method.

To further examine disease-specific reorganization of intrinsic rhythmic control mechanisms related to inter-subsystem coordination, separate multiple linear regression models were fitted to evaluate associations between kinematic rhythm measures derived from the four primary articulators (independent variables) and corresponding acoustic rhythm measures derived from each critical-band envelope (dependent variables). For example, to characterize the kinematic–acoustic relationship underlying prosodic-level rhythmic modulation, the delta modulation depths of the tongue tip, tongue body, lip, and jaw were entered as predictors of the delta modulation depth of each critical-band envelope (100–300, 300–800, 1,000–3,000, and 3,000–8,000 Hz), yielding four multiple linear regression models. The same analyses were performed for syllabic- and sub-syllabic-level rhythmic modulation, regularity of syllable stress, and regularity of intra-syllabic temporal organization by relating the corresponding kinematic and acoustic measures of theta modulation depth, beta/gamma modulation depth, delta-theta PSI, and theta-beta/gamma PSI, respectively.

Based on the regression models, the variance accounted for (VAF) by each kinematic rhythm measure was calculated to index the relative contribution of each articulator to global acoustic rhythmic patterns. Descriptive profiles of kinematic-acoustic relationships were generated from the regression models for each group and compared to identify disease-specific changes in intrinsic rhythmic control mechanisms.

#### 2.7.2 Classification

To evaluate the potential of rhythm and pause measures for differential assessment of motor speech disorders, rhythm measures derived from the acoustic modality, together with all pause measures, were selected for classification analyses. This selection was based on four considerations: (1) as the “end product” of speech production, acoustic measures are theoretically capable of capturing the rhythmic characteristics of the articulatory subsystem; (2) acoustic recording is more feasible for clinical application than kinematic tracking from a practical standpoint; (3) a large set of potentially intercorrelated predictors can increase the risk of overfitting of the classification models trained with a relatively small dataset; and (4) the acoustically derived rhythm measures showed greater overall disease-related changes relative to kinematically derived measures (see Results, Section 3.1).

Classification analyses were performed in two steps. First, canonical discriminant analysis was applied to all acoustically derived rhythm and pause measures to distinguish the ALS, PD, and HC groups. Canonical discriminant analysis is a supervised multivariate statistical method that identifies linear combinations of predictor variables that best distinguish pre-defined groups (Rencher, 1992). Its mathematical foundation is based on the principle of maximizing between-group variability while minimizing within-group variability by solving a generalized eigenvalue problem that identifies directions in the predictor space along which the ratio of between-group to within-group variation is maximized. The resulting discriminant functions represent an orthogonal linear combination of the original predictor variables, with the first function providing the greatest discrimination among groups and subsequent functions explaining progressively smaller amounts of discriminatory information. Individual observations are projected into the canonical space defined by the discriminant functions to generate discriminant scores. This approach enabled (1) visualization of group separation in the canonical space and (2) dimensionality reduction of the predictor space by identifying 10 rhythm and/or pause measures that contributed most strongly to group separation. Predictor importance was quantified by the Euclidean length of each predictor vector in the canonical space, representing its overall association with all canonical discriminant functions.

Second, based on the identified measures, shrinkage discriminant analysis (Friedman, 1989) was performed to classify participants into the ALS, PD, and HC groups. As a regularized variant within the broader family of discriminant analysis, shrinkage discriminant analysis reduces the risk of overfitting and is therefore particularly well suited for smaller datasets. Like linear discriminant analysis, shrinkage discriminant analysis assumes that observations from each class follow a multivariate Gaussian distribution with class-specific mean vectors and a common covariance matrix. Classification is performed by assigning observations to the class with the highest posterior probability based on linear discriminant functions. The major distinction of shrinkage discriminant analysis is the replacement of the empirical covariance matrix with a shrinkage covariance estimator that combines the sample covariance matrix with a structured target matrix.

A repeated nested cross-validation framework was used to implement shrinkage discriminant analysis. The outer loop consisted of 10 independent repetitions of stratified 5-fold cross-validation, yielding 50 independent training-testing splits. Within each outer fold, all predictors were centered and scaled using the training data, and the same pre-processing parameters were subsequently applied to the corresponding test set. For each outer training set, model hyperparameters, including a numeric parameter specifying the degree of shrinkage and a logical parameter specifying the covariance structure (i.e., full or diagonal), were optimized based on the overall accuracy of classification using an inner leave-one-out cross-validation. Following hyperparameter optimization, the best-performing model was refitted using the entire outer training set and evaluated on the corresponding held-out outer test set. Predicted class labels and posterior class probabilities were retained for all test samples across the 10 repetitions and 5 outer folds, resulting in unbiased predictions for every participant. These predictions were subsequently pooled to estimate the mean and 95% confidence intervals of multiclass classification performance metrics, including accuracy, sensitivity, specificity, positive predictive value (PPV), and negative predictive value (NPV), as well as to construct pairwise receiver operating characteristic (ROC) curves.

#### 2.7.3 Associations of rhythm and pause measures with functional outcomes

To evaluate the potential of rhythm and pause measures as global markers of functional speech decline across diseases, all acoustically derived measures were submitted to elastic net regression to predict a functional speech index, which was logarithmically transformed from the SIT-derived speech intelligibility to reduce distributional skewness: log_10_(max(*intelligibility*+1)−*intelligibility*). Elastic net regression is a regularized linear regression method that combines the penalties of lasso (L1) and ridge (L2) regression to improve prediction accuracy and variable selection, particularly in high-dimensional settings (Zou and Hastie, 2005). It minimizes a penalized objective function that includes both L1 and L2 regularization terms, with the relative contribution of the two terms controlled by a mixing parameter α (lasso: α = 1; ridge: α = 0; elastic net: 0 <α <1) and the overall degree of coefficient shrinkage controlled by the regularization parameter λ (larger values corresponding to greater shrinkage). By combining variable selection and coefficient shrinkage, elastic net produces stable, interpretable predictive models while mitigating overfitting.

Model development and validation were performed on centered and scaled predictors using the same repeated nested cross-validation framework described in Section 2.7.2. For each outer training set, the hyperparameters α and λ were optimized based on the root mean squared error (RMSE) using an inner leave-one-out cross-validation. The optimal hyperparameter combination was then used to refit the model on the entire outer training set and evaluate its performance on the corresponding held-out outer test set. Overall model performance was quantified using RMSE, *R*^2^, and mean absolute error (MAE). To further assess model stability and variable importance, regression coefficients from the optimal model in each outer fold were extracted, and the mean coefficient, standard deviation, and selection frequency (i.e., the proportion of outer-fold models in which a coefficient was non-zero) were calculated for each predictor. Finally, a single elastic net model was fitted using the complete dataset with the final hyperparameter combination selected as the most frequently identified α value across the nested cross-validation procedure and the corresponding λ value yielding the lowest RMSE. Predicted values from this model were plotted against the observed outcomes to visually assess model fit.

To examine the potential influence of cognitive impairment on the utility of the rhythm and pause measures as markers of functional speech decline, their associations with the log-transformed MoCA score were evaluated using the same elastic net regression method elaborated above. Markers specific to functional speech decline were expected to predict the functional speech index while exhibiting minimal or no contribution to the cognitive index.

## 3 Results

### 3.1 Disease-specific patterns of rhythmic and pausing disturbances

**Figure 1:**
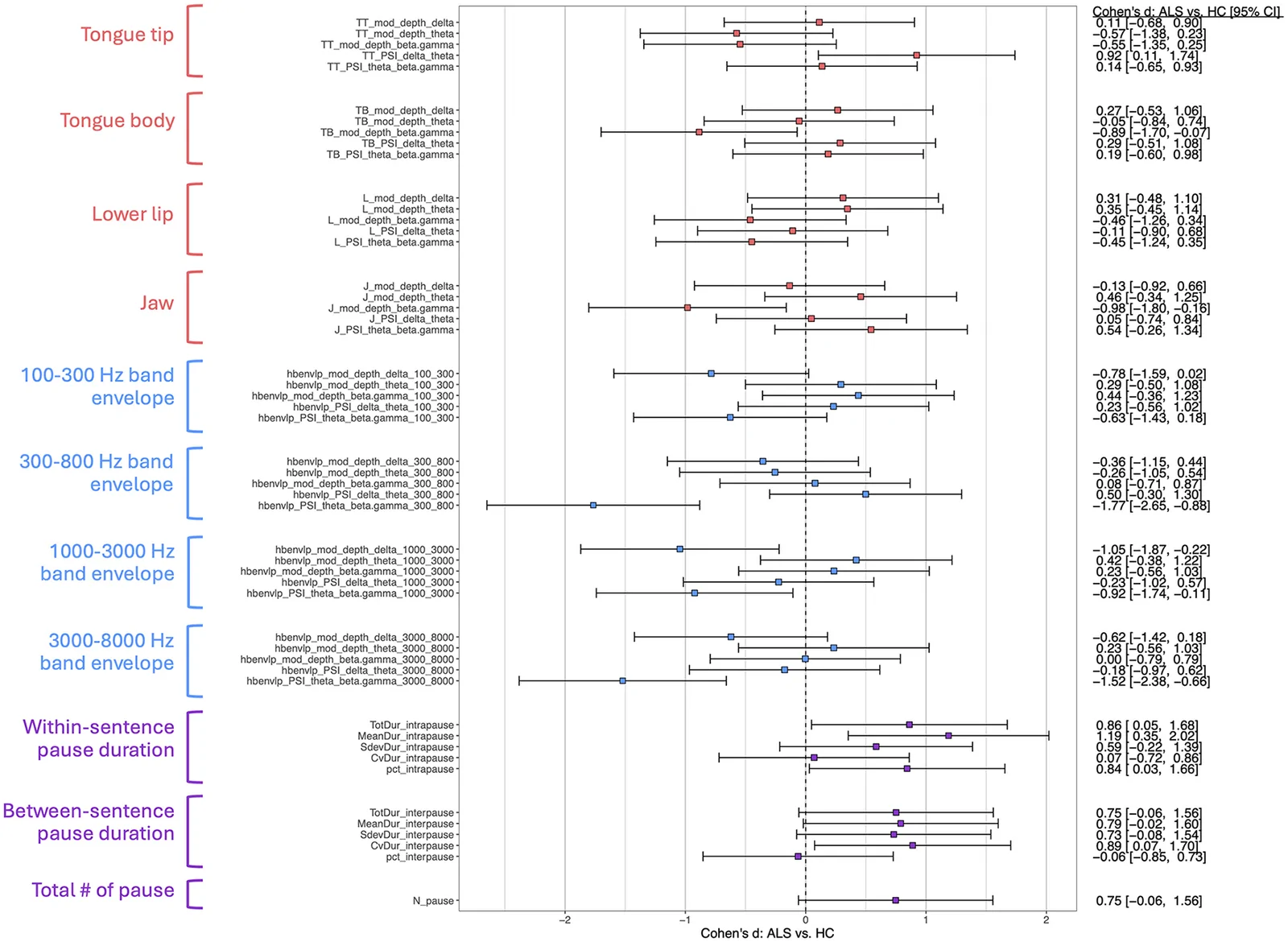
Cohen's d effect sizes for differences in rhythm and pause measures between the amyotrophic lateral sclerosis (ALS) and healthy control (HC) group. Squares and horizontal bars represent the mean effect sizes and corresponding 95% confidence intervals. Squares are color-coded by category: red, kinematic rhythm measures; blue, acoustic rhythm measures; and purple, pause measures. mod_depth_delta, delta modulation depth (prosodic-level modulation); mod_depth_theta, theta modulation depth (syllabic-level modulation); mod_depth_beta.gamma, beta/gamma modulation depth (sub-syllabic-level modulation); PSI_delta_theta, delta-theta phase synchronization index (regularity of syllable stress); PSI_theta_beta.gamma, theta-beta/gamma phase synchronization index (regularity of intra-syllabic temporal organization); TotDur, total duration; MeanDur, mean duration; SdevDur, standard deviation of duration; CvDur, coefficient of variation of duration; pct, percentage of duration.

**Figure 2:**
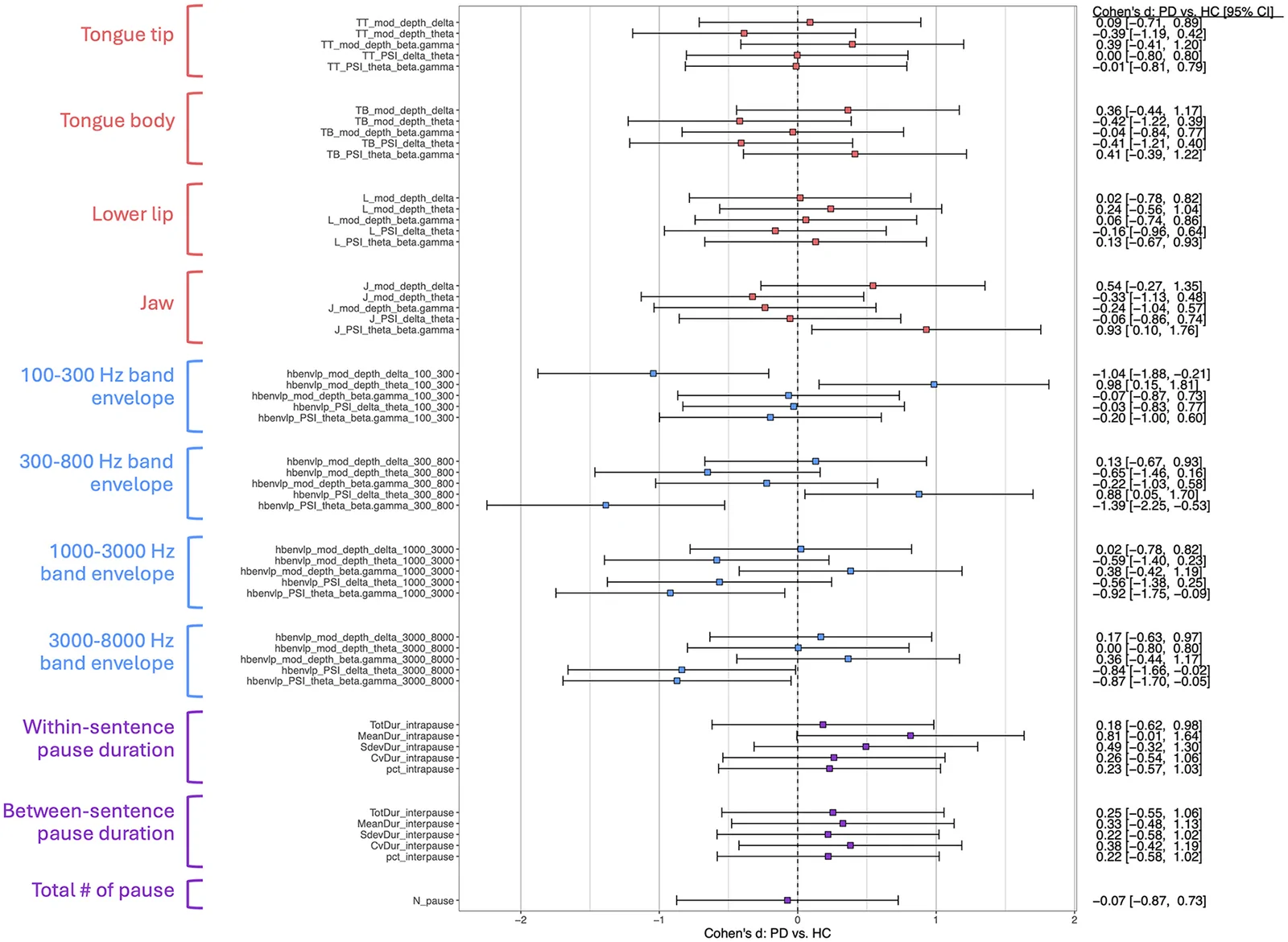
Cohen's d effect sizes for differences in rhythm and pause measures between the Parkinson's disease (PD) and healthy control (HC) group. Squares and horizontal bars represent the mean effect sizes and corresponding 95% confidence intervals. Squares are color-coded by category: red, kinematic rhythm measures; blue, acoustic rhythm measures; and purple, pause measures. mod_depth_delta, delta modulation depth (prosodic-level modulation); mod_depth_theta, theta modulation depth (syllabic-level modulation); mod_depth_beta.gamma, beta/gamma modulation depth (sub-syllabic-level modulation); PSI_delta_theta, delta-theta phase synchronization index (regularity of syllable stress); PSI_theta_beta.gamma, theta-beta/gamma phase synchronization index (regularity of intra-syllabic temporal organization); TotDur, total duration; MeanDur, mean duration; SdevDur, standard deviation of duration; CvDur, coefficient of variation of duration; pct, percentage of duration.

Effect sizes for differences in all rhythm and pause measures between each disease cohort and the HC group are shown in Figures 1, 2. Using an effect size threshold of 0.80 for large effects, the primary disease-related changes in the ALS cohort were characterized by articulatory-driven rhythmic disturbances and altered pausing behaviors both within and between sentences. Rhythmic disturbances were evident in both kinematic measures—including increased delta-theta PSI for the tongue tip (*d* = 0.92, *p* = 0.042) and decreases in beta/gamma modulation depth for the tongue body (*d* = −0.89, *p* = 0.051) and jaw (*d* = −0.98, *p* = 0.059)—and acoustic measures, characterized by reduced delta modulation depth of the 1,000–3,000 Hz band envelope (*d* = −1.05, *p* = 0.020), as well as decreases in theta-beta/gamma PSI across the 300–800 Hz (*d* = −1.77, *p* < 0.001), 1,000–3,000 Hz (*d* = −0.92, *p* = 0.045), and 3,000–8,000 Hz (*d* = −1.52, *p* = 0.0017) band envelopes. Pausing alterations included increases in total duration (*d* = 0.86, *p* = 0.10), mean duration (*d* = 1.19, *p* = 0.017), and proportional duration of within-sentence pauses (*d* = 0.84, *p* = 0.13), as well as increased coefficient of variation of between-sentence pause duration (*d* = 0.89, *p* = 0.10).

The primary disease-related changes in the PD cohort were characterized by respiratory-laryngeal-driven rhythmic disturbances and altered within-sentence pausing behavior. Rhythmic disturbances were pre-dominantly observed in acoustic measures, including decreased delta modulation depth (*d* = −1.04, *p* = 0.044) accompanied by increased theta modulation depth (*d* = 0.98, *p* = 0.063) of the 100–300 Hz band envelope; increased delta-theta PSI in the 300–800 Hz band envelope (*d* = 0.88, *p* = 0.12); decreased delta-theta PSI in the 3,000–8,000 Hz band envelope (*d* = −0.84, *p* = 0.11); and decreased theta-beta/gamma PSI across the 300–800 Hz (*d* = −1.39, *p* = 0.0024), 1,000–3,000 Hz (*d* = −0.92, *p* = 0.045), and 3,000–8,000 Hz band envelopes (*d* = −0.87, *p* = 0.059). The primary pausing alteration was increased mean duration of within-sentence pauses (*d* = 0.81, *p* = 0.080).

**Figure 3:**
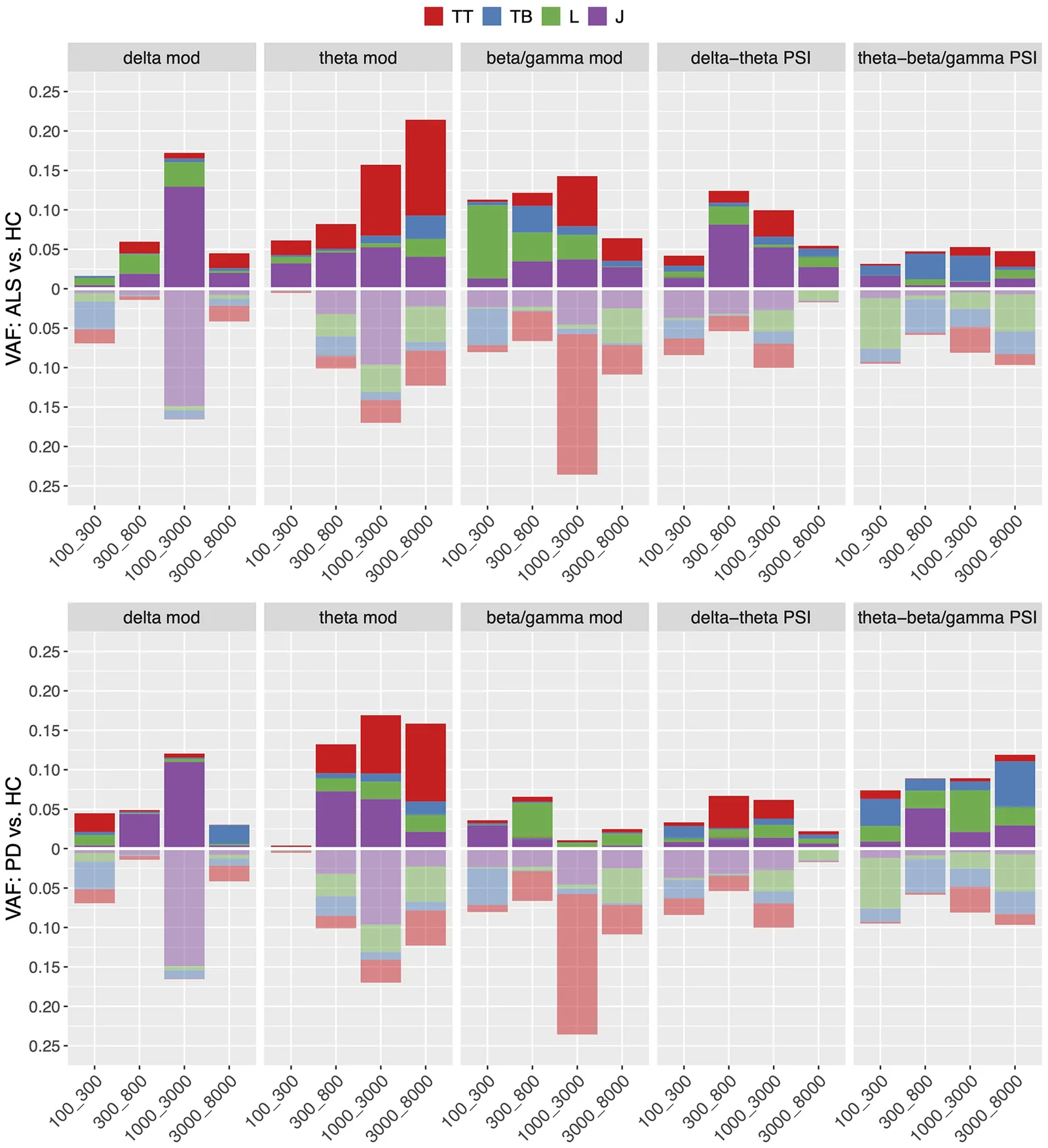
Descriptive profiles of the relationships between kinematic rhythm measures (TT: tongue tip; TB: tongue body; L: lower lip; J: jaw) and their corresponding acoustic rhythm measures. Each profile is organized into five facets corresponding to different aspects of rhythmic modulation: delta modulation depth (delta mod), theta modulation depth (theta mod), beta/gamma modulation depth (beta/gamma mod), delta-theta phase synchronization index (delta-theta PSI), and theta-beta/gamma phase synchronization index (theta-beta/gamma PSI). Within each facet, the relationship between articulatory and acoustic measures is represented by the variance accounted for (VAF) by individual and combined articulatory measures on the y-axis (articulators distinguished by the color of the stacked bars), whereas acoustic measures are organized by critical band on the x-axis. Profiles for the amyotrophic lateral sclerosis (ALS) and Parkinson's disease (PD) groups are displayed above the x-axis, whereas the healthy control (HC) profile, shown for reference, is displayed below the x-axis. Disease and HC profiles are further distinguished by color intensity, with darker shades representing the neurodegenerative disease groups and lighter shades representing the HC group.

Descriptive profiles of the relationships between the kinematic and acoustic rhythm measures are presented in Figure 3. Cross-group comparisons revealed disease-specific changes in rhythmic control mechanisms in both the ALS and PD groups, characterized by altered kinematic–acoustic relationships for different modulation depth measures. As a reference, the HC group demonstrated a distributed pattern of rhythmic modulation that selectively allocates articulatory resources across different levels of the rhythmic hierarchy. Specifically, the jaw served as the primary articulator for prosodic-level modulation, as evidenced by the relationship between delta modulation depths of the jaw and the 1,000–3,000 Hz band envelope. At the syllabic level, all articulators contributed to rhythmic modulation, as reflected by the relationships between theta modulation depths across all four articulators and all three critical-band envelopes associated with articulatory activities (300–800 Hz, 1,000–3,000 Hz, 3,000–8,000 Hz). At the sub-syllabic level, the tongue tip served as the primary articulator for rhythmic modulation, reflected most prominently in the relationship between beta/gamma modulation depths of the tongue tip and the 1,000–3,000 Hz band envelope.

Compared with the HC group, the ALS group revealed a reorganization of articulatory contributions to rhythmic modulation at the syllabic and sub-syllabic levels, characterized by increased contribution of the tongue tip to syllabic-level modulation and decreased contribution of the tongue tip to sub-syllabic-level modulation, accompanied by an increase in lip contribution to sub-syllabic-level modulation. In contrast, the PD group largely preserved kinematic-acoustic relationships at the prosodic and syllabic levels relative to the HC group but exhibited a marked decrease in contributions of all articulators to sub-syllabic-level modulation.

### 3.2 Classification

**Figure 4:**
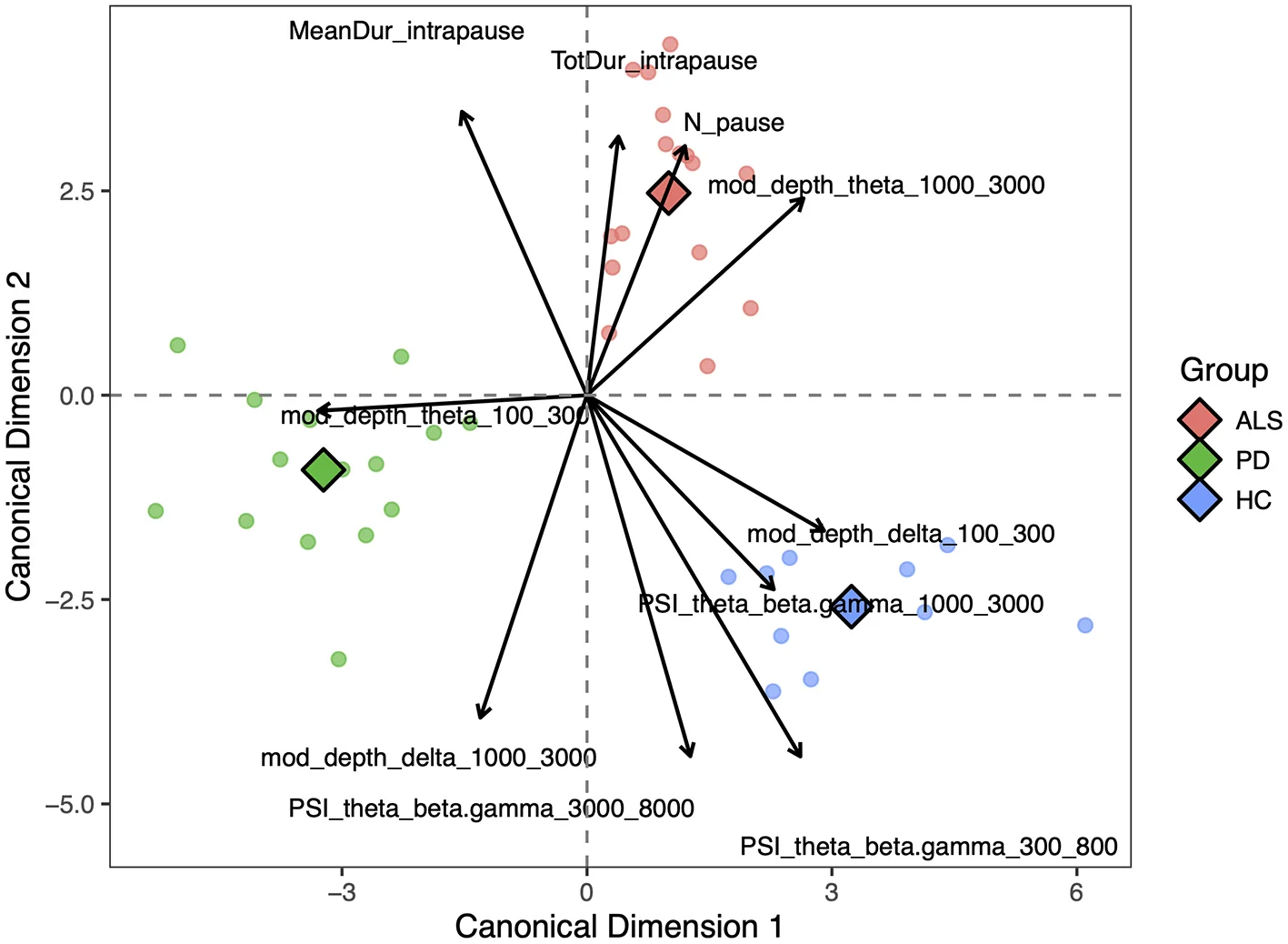
Results of canonical discriminant analysis. In the canonical space, individual observations (dots) and group centroids (diamonds) are color-coded by group: red, amyotrophic lateral sclerosis (ALS); green, Parkinson's disease (PD); and blue, healthy control (HC). Vectors represent the 10 most important rhythm and pause measures driving group separation, with vector orientation denoting the direction of canonical score differences across groups. Rhythm measures include delta modulation depths of the 100–300 Hz and 1,000–3,000 Hz band envelopes (mod_depth_delta_100_300, mod_depth_delta_1000_3000), theta modulation depths of the 100–300 Hz and 1,000–3,000 Hz band envelopes (mod_depth_theta_100_300, mod_depth_theta_1000_3000), and theta-beta/gamma phase synchronization indices across the 300–800 Hz, 1,000–3,000 Hz, and 3,000–8,000 Hz band envelopes (PSI_theta_beta.gamma_300_800, PSI_theta_beta.gamma_1000_3000, PSI_theta_beta.gamma_3000_8000). Pause measures included the total duration and mean duration of within-sentence pauses (TotDur_intrapause, MeanDur_intrapause), as well as the total number of pauses throughout the passage (N_pause).

The results of the canonical discriminant analysis are shown in Figure 4, which identified seven rhythm measures and three pause measures as the most important contributors to group separation. Rhythm measures included delta modulation depths of the 100–300 Hz and 1,000–3,000 Hz band envelopes, theta modulation depths of the 100–300 Hz and 1,000–3,000 Hz band envelopes, and theta-beta/gamma PSI across the 300–800 Hz, 1,000–3,000 Hz, and 3,000–8,000 Hz band envelopes. Pause measures included the total duration and mean duration of within-sentence pauses, as well as the total number of pauses throughout the passage.

Among these measures, the separation of both disease groups from the HC group was primarily driven by two sets of measures: reduced theta-beta/gamma PSI across the 300–800 Hz, 1,000–3,000 Hz, and 3,000–8,000 Hz band envelopes and increased mean duration of within-sentence pauses. In addition, the ALS group exhibited several disease-specific patterns, including decreased delta modulation depth and increased theta modulation depth of the 1,000–3,000 Hz band envelope, as well as increased total duration of within-sentence pauses and a greater total number of pauses. The PD group also exhibited disease-specific patterns, characterized by decreased delta modulation depth and increased theta modulation depth of the 100–300 Hz band envelope.

**Figure 5:**
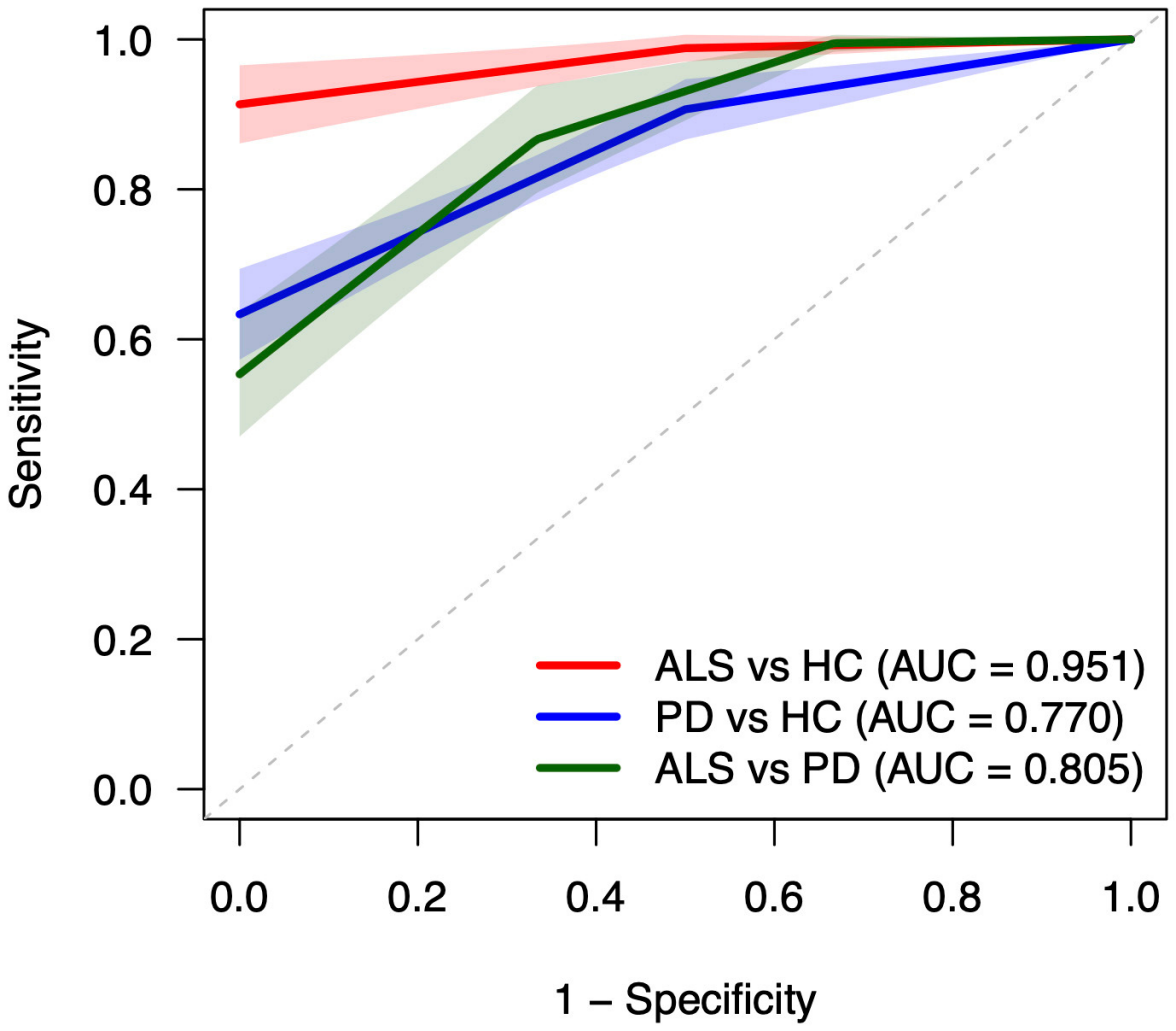
Receiver Operating Characteristic (ROC) curves and corresponding area under the curve (AUC) values for pairwise classification among the amyotrophic lateral sclerosis (ALS), Parkinson's disease (PD), and healthy control (HC) groups. Solid lines and shaded regions represent the mean ROC curves and their corresponding 95% confidence intervals, respectively, estimated from the repeated nested cross-validation framework based on shrinkage discriminant analysis.

Collectively, the 10 selected rhythm and pause measures accurately distinguished all three groups, as demonstrated by the pairwise ROC curves in Figure 5 and the multiclass classification performance metrics in Table 4. Across the three groups, the classifier achieved a mean accuracy of 0.77, sensitivity of 0.73, specificity of 0.83, PPV of 0.86, and NPV of 0.75.

**Table 4:** Multiclass classification performance of shrinkage discriminant analysis, reported as the mean with 95% confidence intervals (in brackets) estimated from repeated nested cross-validation.

Performance Metric	ALS vs. HC	PD vs. HC	ALS vs. PD
Accuracy	0.87 [0.84, 0.90]	0.76 [0.74, 0.78]	0.67 [0.64, 0.70]
Sensitivity	0.80 [0.76, 0.85]	0.75 [0.72, 0.79]	0.63 [0.60, 0.65]
Specificity	0.98 [0.95, 1.00]	0.78 [0.74, 0.82]	0.75 [0.71, 0.79]
PPV	0.98 [0.94, 1.00]	0.84 [0.82, 0.87]	0.77 [0.74, 0.81]
NPV	0.84 [0.81, 0.88]	0.76 [0.71, 0.80]	0.66 [0.64, 0.68]

ALS, amyotrophic lateral sclerosis; PD, Parkinson's disease; HC, healthy controls; PPV, positive predictive value; NPV, negative predictive value.

### 3.3 Associations of rhythm and pause measures with functional outcomes

**Figure 6:**
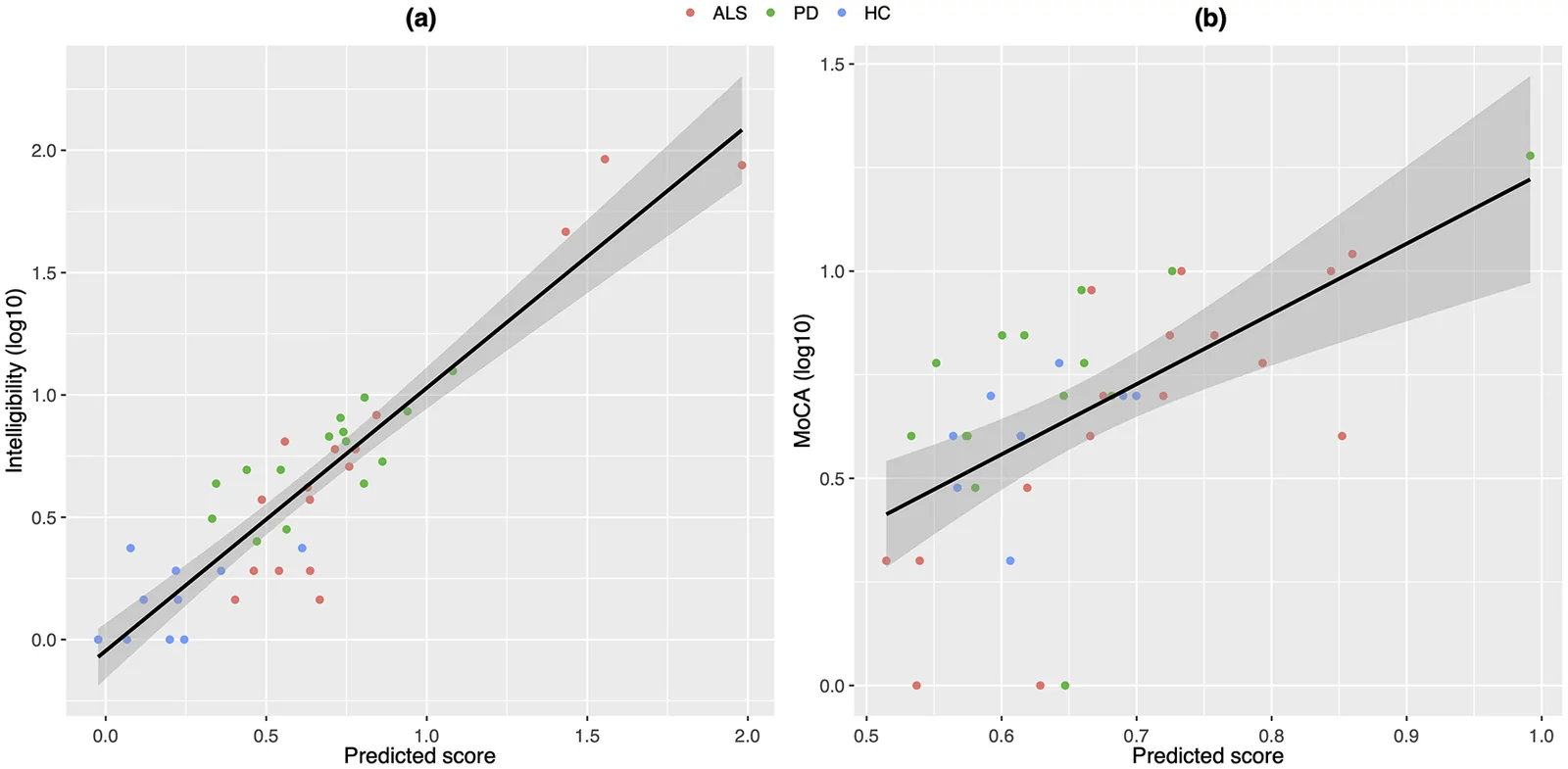
Relationships between observed and predicted values for **(a)** speech intelligibility and **(b)** Montreal Cognitive Assessment (MoCA) scores. Both outcomes were log-transformed prior to analysis. Individual observations are color-coded by group: red, amyotrophic lateral sclerosis (ALS); green, Parkinson's disease (PD); and blue: healthy control (HC). Black lines and the surrounding shared regions represent the mean regression lines and corresponding 95% confidence intervals, respectively, estimated by from repeated nested cross-validation of elastic net regression.

The rhythm and pause measures were selectively associated with speech intelligibility. As shown in Table 5, the primary contributors to speech intelligibility decline, represented by measures selected in more than 50% of the fitted elastic net regression models, included (1) reduced delta modulation depths of the 100–300 Hz, 1,000–3,000 Hz, and 3,000–8,000 Hz band envelopes, (2) increased theta modulation depths of the 100–300 Hz and 3,000–8,000 Hz band envelopes, (3) reduced theta-beta/gamma PSI across the 300–800 Hz, 1,000–3,000 Hz, and 3,000–8,000 Hz bands, (4) increased total duration, mean duration, and variability of within-sentence pauses, and (5) increased variability and reduced proportional duration of between-sentence pauses. The relationship between the predicted outcome based on these selected measures and the log-transformed speech intelligibility is shown in Figure 6a, with R^2^ = 0.39 (95% CI: 0.32–0.46), RMSE = 0.40 (95% CI: 0.36–0.44), MAE = 0.32 (95% CI: 0.28–0.35).

**Table 5:** Mean coefficient, standard deviation (SD), and selection frequency (SelFreq) of rhythm and pause measures estimated from repeated nested cross-validation of elastic net regression models predicting log-transformed speech intelligibility and Montreal Cognitive Assessment (MoCA) scores.

Predictor	Intelligibility			MoCA score		
	Mean	SD	SelFreq	Mean	SD	SelFreq
hbenvlp_mod_depth_delta_100_300	−0.042	0.030	0.90	−0.005	0.028	0.16
hbenvlp_mod_depth_delta_300_800	0.016	0.072	0.26	0.003	0.020	0.06
hbenvlp_mod_depth_delta_1000_3000	−0.029	0.078	0.74	−0.003	0.021	0.04
hbenvlp_mod_depth_delta_3000_8000	−0.034	0.043	0.80	0.017	0.074	0.12
hbenvlp_mod_depth_theta_100_300	0.024	0.034	0.72	0.002	0.022	0.30
hbenvlp_mod_depth_theta_300_800	−0.017	0.059	0.32	−0.008	0.026	0.12
hbenvlp_mod_depth_theta_1000_3000	0.020	0.062	0.36	−0.008	0.034	0.12
hbenvlp_mod_depth_theta_3000_8000	0.034	0.034	0.84	−0.001	0.022	0.08
hbenvlp_mod_depth_beta.gamma_100_300	−0.010	0.057	0.38	−0.001	0.014	0.06
hbenvlp_mod_depth_beta.gamma_300_800	−0.001	0.030	0.26	0.006	0.026	0.06
hbenvlp_mod_depth_beta.gamma_1000_3000	−0.009	0.045	0.18	0.011	0.022	0.44
hbenvlp_mod_depth_beta.gamma_3000_8000	0.012	0.047	0.14	0.001	0.006	0.06
hbenvlp_PSI_delta_theta_100_300	−0.010	0.034	0.26	−0.003	0.014	0.14
hbenvlp_PSI_delta_theta_300_800	0.003	0.031	0.28	−0.015	0.031	0.44
hbenvlp_PSI_delta_theta_1000_3000	−0.022	0.043	0.52	−0.023	0.021	0.76
hbenvlp_PSI_delta_theta_3000_8000	−0.008	0.026	0.56	−0.003	0.010	0.10
hbenvlp_PSI_theta_beta.gamma_100_300	−0.018	0.033	0.68	−0.013	0.026	0.30
hbenvlp_PSI_theta_beta.gamma_300_800	−0.059	0.039	0.94	0.003	0.014	0.12
hbenvlp_PSI_theta_beta.gamma_1000_3000	−0.062	0.036	0.92	0.006	0.015	0.30
hbenvlp_PSI_theta_beta.gamma_3000_8000	−0.038	0.025	0.88	0.002	0.005	0.14
TotDur_intrapause	0.020	0.060	0.82	0.000	0.017	0.10
MeanDur_intrapause	0.066	0.062	0.98	0.006	0.033	0.12
SdevDur_intrapause	0.043	0.058	0.84	−0.003	0.024	0.06
CvDur_intrapause	0.029	0.053	0.80	0.004	0.017	0.06
pct_intrapause	−0.008	0.039	0.08	0.085	0.038	1.00
TotDur_interpause	0.004	0.020	0.14	0.007	0.026	0.24
MeanDur_interpause	0.006	0.029	0.24	0.020	0.036	0.70
SdevDur_interpause	0.020	0.052	0.30	−0.003	0.019	0.02
CvDur_interpause	0.017	0.081	0.76	−0.004	0.019	0.06
pct_interpause	−0.061	0.071	0.92	−0.004	0.031	0.10
N_pause	−0.003	0.036	0.50	0.002	0.007	0.12

Predictors selected in no less than 70% of the fitted models (SelFreq ≥ 0.70) are highlighted in red.

Increased proportional duration of within-sentence pauses emerged as the primary contributor to cognitive decline, as indexed by reduced MoCA score, accompanied by two additional but less frequently selected contributors: increased mean duration of between-sentence pauses and reduced delta-theta PSI in the 1,000–3,000 Hz band envelope. The relationship between the predicted outcome based on these selected measures and the log-transformed MoCA score is shown in Figure 6b, with R^2^ = 0.26 (95% CI: 0.20–0.32), RMSE = 0.28 (95% CI: 0.25–0.30), MAE = 0.21 (95% CI: 0.19–0.23).

## 4 Discussion

This study employed a multimodal analytic framework to derive a set of interpretable, objective measures characterizing rhythmic and pausing behaviors during a passage reading task in two neurodegenerative disease cohorts: ALS and PD. Based on these measures, both disease cohorts exhibited disrupted prosodic rhythm modulation and reduced regularity of intra-syllabic temporal organization. However, the physiological substrates underlying prosodic rhythm disruptions differed, with ALS-related disruption driven primarily by articulatory impairment and PD-related disruption driven pre-dominantly by respiratory-laryngeal impairment. Pausing behaviors also differed between diseases: the ALS cohort showed widespread alterations in pausing both within and across sentences, whereas the PD cohort primarily revealed disturbances in within-sentence pausing. Collectively, these rhythm and pausing measures (1) accurately differentiated both the ALS and PD cohorts from healthy control speakers, as well as from each other, and (2) were selectively associated with functional speech performance across diseases. These findings support the potential utility of the rhythm and pause measures as objective markers to facilitate differential assessment of motor speech disorders and as global indicators of functional speech decline, thereby advancing measurement-based care in neurodegenerative diseases.

### 4.1 Disease-specific patterns of rhythmic and pausing disturbances

Consistent with our hypothesis, disease-specific patterns of rhythmic and pausing disturbances were identified. As shown in Figure 1, in the ALS cohort, rhythmic disturbances were manifested in kinematic rhythm measures for individual articulators, including (1) increased delta-theta PSI for the tongue tip, which may be attributed to reduced dynamic flexibility or compensatory stabilization that leads to stronger coupling between slow prosodic organization and syllabic-level tongue tip movement timing, and (2) reduced beta/gamma modulation depths of the tongue body and jaw, likely reflecting impaired fine-grained articulatory movements for modulating fast rhythms, consistent with prior findings in Rong and Heidrick (2024). ALS-related rhythmic disturbances were also evident in acoustic envelope modulation patterns, characterized by (1) reduced delta modulation depth of the 1,000–3,000 Hz band envelope, indicative of articulatory-driven disruption of slow prosodic rhythm, and (2) reduced theta-beta/gamma PSI across the 300–800 Hz, 1,000–3,000 Hz, and 3,000–8,000 Hz band envelopes, suggesting weakened coupling between syllabic and sub-syllabic timing and, consequently, reduced regularity of intra-syllabic temporal organization. Compared with prior studies that evaluated rhythmic control during short sentence reading and unstructured picture description tasks (Rong and Heidrick, 2024; Rong and Liston, 2025), the passage reading task evaluated in the present study appears to provide a more robust context to capture prosodic rhythm disturbances.

In addition to the changes in kinematic and acoustic rhythm measures, the relationship between these measures was also altered in the ALS cohort (Figure 3). The most prominent alteration was a shift in tongue tip engagement from its role as the primary articulator for sub-syllabic-level modulation in the HC group toward enhanced engagement in syllabic-level modulation. This shift likely arises from impaired fine motor control of the tongue tip—an articulator reported to be among the earliest and most severely affected in ALS (DePaul et al., 1988)—restricting its capacity to generate fine-grained movements required for fast sub-syllabic rhythms. As a potentially adaptive response, individuals with ALS may reallocate articulatory resources based on their differential impairments, shifting the functional role of the tongue tip from sub-syllabic rhythmic modulation toward slower, syllable-centered rhythmic modulation, while increasingly recruiting a relatively less impaired articulator—the lower lip—to support faster, sub-syllabic rhythmic modulation. Taken together, these alterations are interpreted to reflect differential impairments of the articulators and a putative reorganization of the articulatory subsystem to support hierarchical rhythmic modulation while accommodating and mitigating these impairments.

Pausing alterations in the ALS cohort were characterized by increased total duration, mean duration, and proportional duration of within-sentence pauses, as well as increased variability of between-sentence pause duration. Longer and more variable pauses have also been previously reported in ALS (Green et al., 2018, 2004; Yunusova et al., 2016). The present study further extends prior findings by distinguishing between pauses within and between sentence boundaries and demonstrating that the two types of pauses were differentially affected in ALS: the increase in pause duration primarily arose from within-sentence pauses, whereas the increase in pause variability was driven pre-dominantly by between-sentence pauses. Notably, the total duration and mean duration of within-sentence pauses, as well as the variability of between-sentence pauses were identified as correlates of speech intelligibility, whereas the proportional duration of within-sentence pauses was associated with the MoCA score (Table 5). Integrating these findings suggests that the differential changes in within- and between-sentence pauses observed in the ALS cohort may reflect the combined effects of respiratory and cognitive-linguistic impairments.

In the PD cohort, rhythmic disturbances were primarily reflected in acoustic envelope modulation patterns in the 100–300 Hz band, while kinematic rhythm measures were generally less affected than in the ALS cohort (Figure 2). These observations are consistent with the hallmark features of Parkinsonian dysarthria, which is classified as respiratory-laryngeal dominant in the classic auditory-perceptual evaluation framework (Darley et al., 1969a,b). The finding of reduced delta modulation depth accompanied by increased theta modulation depth in the 100–300 Hz band envelope suggests a shift away from a typical prosodic stress–based rhythmic modulation paradigm—consistent with English as a stress-timed language—toward diminished prosodic-level rhythmic modulation and enhanced syllabic-level rhythmic modulation of intonation. These changes would reduce the prosodic prominence of stressed syllables, consistent with the percept of reduced stress in PD (Darley et al., 1969a,b). In addition, delta-theta PSI demonstrated an increase in the 300–800 Hz band and a decrease in the 3,000–8,000 Hz band in the PD cohort. Given that the 300–800 Hz band contains spectral information related to the formants of syllable nuclei, increased delta-theta PSI in this band can be interpreted as reflecting reduced flexibility in temporal organization of segmental articulatory events within prosodic structure, which may be attributed to rigidity and/or impaired internal timing control in PD. The physiological basis of the decrease in delta-theta PSI in the 3,000–8,000 Hz band remains less clear but may reflect compensatory adaptations to changes in the lower-frequency range. In contrast, theta-beta/gamma PSI was decreased across the 300–800 Hz, 1,000–3,000 Hz, and 3,000–8,000 Hz bands—a pattern consistent with that observed in the ALS cohort—suggesting that reduced regularity of intra-syllabic temporal organization may represent a shared marker of motor speech impairment across diseases.

The relationship between kinematic and acoustic rhythm measures revealed globally reduced articulatory contributions to beta/gamma acoustic envelope modulation across spectral bands (Figure 3). Unlike ALS, which results from the degeneration of the pyramidal system and differentially affects craniofacial motor neurons and their associated articulators, PD arises from the degeneration of the extrapyramidal system, which is involved in global motor scaling and movement regulation. Motor scaling deficits in PD are therefore expected to affect all articulators, resulting in bradykinesia that is most prominently expressed in fine-grained articulatory movements related to fast sub-syllabic rhythms. Such fine-grained motor deficits provide a potential explanation for the observed global reduction in articulatory contributions to beta/gamma modulation.

PD-related pausing alteration was marked by increased mean duration of within-sentence pauses, whereas between-sentence pauses remain relatively unaffected. Given that mean within-sentence pause duration was identified as a correlate of speech intelligibility (Table 5), the pausing alteration in PD is mostly likely a manifestation of respiratory impairment rather than cognitive-linguistic impairment.

### 4.2 Rhythm and pause measures as objective markers to enhance differential assessment of motor speech disorders

The disease-related rhythmic and pausing disturbances provide valuable insights for the detection and differential characterization of motor speech impairments related to distinct etiologies. Specifically, measures that capture shared disturbances across diseases, including three rhythm measures (i.e., theta-beta/gamma PSI within the 300–800 Hz, 1,000–3,000 Hz, and 3,000–8,000 Hz bands) and one pause measure (i.e., mean within-sentence pause duration), differentiated both disease cohorts from the HC group, as demonstrated in Figure 4. Measures capturing ALS-specific disturbances, including delta and theta modulation depths of the 1,000–3,000 Hz envelope, total duration of within-sentence pauses, along with total number of pauses, distinguished the ALS cohort from both PD and HC groups. Measures capturing PD-specific disturbances, including delta and theta modulation depths of the 100–300 Hz envelope, differentiated the PD cohort from both ALS and HC groups. Collectively, these rhythm and pause measures enabled accurate classification of all participants into the ALS, PD, and HC groups with promising classification performance, as demonstrated by both the performance metrics in Table 4 (accuracy = 0.67–0.87) and the ROC curves in Figure 5 (AUC = 0.77–0.95).

From a translational perspective, the rhythm and pause measures identified above provide clinically applicable, objective markers with strong potential for differential assessment of motor speech disorders. These markers are derived from a clinically implementable modality (i.e., acoustics) using pre-dominantly automated analytic procedures while retaining clinical and physiological interpretability. Throughout the workflow of this study, including data collection, signal processing, marker development, and data-driven modeling (i.e., classification and regression), both the hardware requirement for acoustic data collection and the computational demands of signal processing, feature extraction, marker development, and machine learning analyses are minimal. The only manual step in the current workflow is the segmentation of pauses from the acoustic recordings. However, prior work has demonstrated the feasibility of algorithmic pause estimation in individuals with motor speech disorders (Green et al., 2004), providing a more efficient alternative to manual pause segmentation. Future research should focus on optimizing algorithmic parameters to improve the accuracy and robustness of automated pause detection across neurodegenerative etiologies and varying levels of motor speech impairment, thereby enabling a fully automated workflow and facilitating its integration into routine clinical use.

The measures derived in this study integrate the strengths of traditional handcrafted measures, which are interpretable but labor-intensive and expertise-dependent, and contemporary computational features, which can be efficiently extracted but often lack transparency and explainability. Moreover, compared with the conventional auditory-perceptual evaluation, the analytic framework in this study offers the advantage of identifying specific physiological substrates underlying clinical manifestations. For example, while both ALS and PD exhibited prosodic and rhythmic disturbances at the clinical level, such disturbances were underpinned by different physiological substrates (Figures 1, 2). These substrates constituted key contributors to the separation between the two disease cohorts (Figure 4), highlighting the added value of the analytic framework over conventional auditory-perceptual methods for differential assessment of motor speech disorders.

### 4.3 Rhythm and pause measures as objective indicators of functional speech decline across neurodegenerative motor speech disorders

The majority of rhythm and pause measures that contributed to the detection and differentiation of motor speech impairments in ALS and PD also demonstrated meaningful associations with functional speech performance across diseases. As shown in Table 5, reduced speech intelligibility was linked to (1) reduced delta modulation depth and increased theta modulation depth of the 100–300 Hz envelope (observed primarily in PD; Figure 2) and reduced delta modulation depth of the 1,000–3,000 Hz envelope (observed primarily in ALS; Figure 1), collectively reflecting a putative reorganization of hierarchical rhythmic modulation between prosodic and syllabic levels; (2) reduced regularity of intra-syllabic temporal organization across the 300–800 Hz, 1,000–3,000 Hz, and 3,000–8,000 Hz bands (observed in both ALS and PD); and (3) pausing alterations, reflected by increased total duration of within-sentence pauses and increased variability of between-sentence pauses (observed primarily in ALS), as well as increased mean duration of within-sentence pauses (observed across ALS and PD). These, along with several additional measures (i.e., delta and theta modulation depths of the 3,000–8,000 Hz band envelope, variability of within-sentence pause duration, and proportional duration of between-sentence pauses), constituted key contributors to speech intelligibility decline across diseases. Importantly, none of these measures overlapped with the correlates of the MoCA score, indicating their relative robustness to influences from cognitive-linguistic impairments, which often cooccur with and interact alongside motor speech impairments to jointly shape communicative outcomes in neurodegenerative diseases. Taken together, these findings support the potential of the selected rhythm and pause measures as objective indicators of functional speech decline. Clinically, these measures may enable more targeted intervention while providing quantitative feedback on intervention outcomes by capturing subtle yet meaningful subclinical changes that may not be detectible in standardized functional outcomes, ultimately advancing measurement-based care in neurodegenerative diseases.

### 4.4 Limitations and future directions

Several limitations of this study should be acknowledged. In the marker development phase, multiple potential confounding factors may influence the performance of the rhythm and pause measures as objective markers of motor speech impairment. These factors include (1) biological variables, such as imbalanced sex distributions of the sample and age-related changes in speech production; (2) clinical variables, including disease duration and severity, medication status, and participation in clinical trials; and (3) technical variables, such as differences in recording equipment and acquisition specifications. The extent to which these factors influence marker performance remains unclear and warrants systematic investigation in future studies.

In the data-driven model development phase, the relatively small sample size and the lack of external validation may increase the risk of overfitting, thereby limiting the generalizability of the classification and regression models. To mitigate this risk, we employed a repeated nested cross-validation framework that separates hyperparameter tuning from model training and performance evaluation while repeatedly resampling the data to reduce dependence on any single train–test split. Averaging performance across multiple nested resampling iterations provides more stable and less optimistic estimates of model performance than a single cross-validation procedure, thereby improving the robustness of model evaluation. Nevertheless, external validation using independent cohorts remains essential to confirm the generalizability and clinical applicability of the models.

Heterogeneity is a hallmark feature of neurodegenerative diseases (Brettschneider et al., 2015; Walker, 2016). Variability in disease onset in ALS (e.g., bulbar vs. spinal) and dominant symptom presentation in PD (e.g., bradykinesia/rigidity vs. tremor), along with other clinicopathological features, may give rise to distinct phenotypes with different manifestations of motor speech impairment. However, due to the relatively small sample size and the unavailability of comprehensive, standardized clinical records for all participants resulting from the diverse recruitment sources in this study, all analyses were conducted at the cohort level without further participant stratification. With a larger sample, subgroups with distinct rhythmic and pausing profiles may emerge from each disease cohort. Future studies should investigate the feasibility of using the rhythm and pause measures derived in this study for patient phenotyping and stratification to guide individualized care for neurodegenerative motor speech disorders.

Compared with prior studies that applied similar rhythmic analysis to different speech tasks (e.g., short sentence reading, unstructured picture description) (Rong and Heidrick, 2024; Rong and Liston, 2025), various differences in rhythmic characteristics were identified in the present study, suggesting that rhythmic control is task-dependent and that tasks with differing motor and cognitive-linguistic demands may vary in their sensitivity to rhythmic disturbances at prosodic, syllabic, and sub-syllabic levels. For example, the structured passage reading task employed in this study appears to be particularly sensitive to prosodic-level rhythmic disturbances; the short sentence reading task in Rong and Liston (2025) appears more susceptible to fast sub-syllabic-level rhythmic disturbances; and the unstructured picture description task in Rong and Heidrick (2024) appears sensitive to both syllabic- and sub-syllabic-level rhythmic disturbances. A detailed characterization of the interaction between task demands and hierarchical rhythmic modulation could provide useful insights for designing rhythm-based assessment protocols optimized for motor speech evaluation. In addition to task, language itself has intrinsic rhythmic characteristics that may influence the performance of the rhythm measures. This study investigated English, a language traditionally classified as stress-timed, whereas syllable-timed and tonal languages exhibit systematically different rhythmic organizations. Consequently, the present findings may not generalize directly to other languages. Future studies should investigate how speech task and language interact with rhythmic organization and evaluate the cross-linguistic generalizability of the proposed rhythm measures.

Among all pause measures examined in this study, within- and between-sentence pausing behaviors were differentially associated with motor speech and cognitive-linguistic impairments. As shown in Table 5, increased proportional duration of between-sentence pauses was correlated with better speech intelligibility, whereas increased proportional duration of within-sentence pauses was associated with lower MoCA scores. The positive association between relative duration of between-sentence pauses and speech intelligibility may reflect strategic breath management to support preparation for subsequent sentence production. In contrast, the negative association between relative duration of within-sentence pauses and global cognitive performance may reflect cognitive-linguistic deficits in lexical retrieval and syntactic processing, particularly when these pauses occur at grammatically inappropriate locations, with downstream effects on motor planning and programming. While a detailed linguistic analysis of pausing behaviors is beyond the scope of this study, it may provide valuable insights into the differentiation and profiling of motor-driven vs. cognitive-linguistically driven pausing alterations.

## 5 Conclusions

Using a mechanistically informed analytic framework, this study derived interpretable objective measures from non-invasive orofacial motion tracking and acoustic recordings to characterize the physiological substrates underlying hierarchical rhythmic modulation and pausing behaviors during a passage reading task. Based on these measures, both shared and distinct patterns of rhythmic and pausing disturbances were identified from two neurodegenerative diseases, ALS and PD:

- Shared patterns included reduced regularity of intra-syllabic temporal organization and increased mean duration of within-sentence pauses.
- ALS-specific patterns included an articulatory-driven rhythmic reorganization, characterized by reduced prosodic-level modulation and increased syllabic-level modulation of the 1,000–3,000 Hz band acoustic envelope, as well as increased variability of between-sentence pauses.
- PD-specific patterns were represented by a respiratory-laryngeal driven rhythmic reorganization, characterized by reduced prosodic-level modulation and increased syllabic-level modulation of the 100–300 Hz band acoustic envelope.

The identified disease-specific impairment patterns are physiologically meaningful, which can be interpreted as reflecting neuromuscular weakness in ALS pre-dominantly affecting articulatory behaviors and deficits in motor scaling and internal timing control in PD primarily manifested in voicing and intonation behaviors. The combination of measures capturing both shared and disease-specific rhythmic and pausing disturbances demonstrated promising discriminatory performance for detecting and differentiating motor speech impairments in ALS and PD, while also exhibiting meaningful relationships with functional speech decline across diseases. Given their minimal hardware requirement and computational demands, the proposed rhythm and pause measures hold strong potential as clinically applicable, objective makers to complement existing clinical methods, supporting more accurate differential diagnosis, targeted intervention, and measurement-based care for neurodegenerative motor speech disorders, in alignment with the broader framework of precision neurology.

## Data Availability

The datasets presented in this article are not readily available because these datasets contain potentially identifiable information about the research participants. Raw data will be made available in a de-identified format upon reasonable request and appropriate data sharing agreement. Requests to access the datasets should be directed to Panying Rong, prong@ku.edu.
