# Supplementary material for: A mechanistically informed framework of hierarchical temporal speech organization for objective differential assessment of motor speech disorders in neurodegenerative diseases
Source: Front Neurosci. 2026 Sep 4;20:1907826. doi: 10.3389/fnins.2026.1907826 (PMC13587793; doi:10.3389/fnins.2026.1907826)
Supplement: Supplementary file 1 [file Table_1.docx]

**Supplemental Materials**

Table S1. Checklist for auditory-perceptual features.

| **Dimension** | **Feature** |
| --- | --- |
| Articulatory | Imprecise consonant  Distorted vowels  Irregular articulatory breakdown  Articulatory groping  Increased errors with increased rate  Prolonged phonemes  Repeated phonemes |
| Phonatory | Harsh voice  Hoarse voice  Breathy voice  Strained-strangled voice  Voice stoppages  Pitch level  Pitch breaks  Voice tremor  Diplophonia |
| Resonatory | Hypernasality  Hyponasality  Nasal emission  Weak pressure consonants |
| Respiratory | Forced inspiration-expiration  Audible inspiration  Grunt at the end of expiration |
| Rate | Reduced rate overall  Increased rate overall |
| Rhythm | Rushed of speech  Increased rate in segments  Variable rate |
| Stress | Reduced stress  Excess and equal stress |
| Prosody (vocal intensity) | Monoloudness  Excess loudness variation  Alternating loudness  Loudness decay |
| Prosody (vocal pitch) | Monopitch |
| Prosody (phrasing) | Short phrases  Prolonged intervals  Inappropriate silences |
